# The interference of voice change on structural vocal cords lesions

**DOI:** 10.1016/S1808-8694(15)31070-3

**Published:** 2015-10-22

**Authors:** Mônica Alcantara de Oliveira Santos, José Marcos Pechula Moura, André de Campos Duprat, Henrique Olival Costa, Bianca Benatti de Azevedo

**Affiliations:** 1 Otorhinolaryngologist, taking a postgraduate course at the Sao Paulo Santa Casa de Misericordia.; 2 Otorhinolaryngologist trained at the Sao Paulo Santa Casa de Misericordia.; 3 Doctor, Assistant Professor at the Sao Paulo Santa Casa Medical School, Assistant doctor of the Otorhinolaryngology Department of the Sao Paulo Santa Casa de Misericordia.; 4 Doctor, Assistant Professor at the Sao Paulo Santa Casa Medical School.; 5 Speech therapist, voice specialist trained at the Sao Paulo Santa Casa de Misericordia. Sao Paulo Santa Casa de Misericordia.

**Keywords:** structural lesions, voice change, vocal cords

## Summary

Alongitudinal cohort study Introduction: Voice change may be defined as a group of changes in voice pattern that take place between childhood and puberty. During this period some vocal cord lesions (specifically cysts and nodules) may undergo transformation. **Aim:** To evaluate changes in vocal cord structural lesions following voice changes. **Material and method:** All laringoscopic exams made at the Sao Paulo Santa Casa de Misericordia between 1997 and 2002 of children aged below 10 years with structural lesions were reevaluated. Children whose voice had already changed repeated the exam and answered a questionary about voice change. **Results:** Eleven children were studied. Observation showed that hoarseness was significantly decreased after voice change, and that lesions revealed modifications. Thickenedlike lesions were reabsorved, and protrusion-like lesions underwent modification but did not disappear. **Conclusion:** The definition of lesions and subsequent modifications after voice change are important to define the correct approach to children presenting hoarseness.

## INTRODUCTION

Voice change may be defined as a set of changes in the pattern of voice that takes place between childhood and puberty. It is expected between ages 12 and 14 years in women and between ages 13 and 15 in men.^1^ These changes are characterized not only by increased intensity but also changes in the fundamental frequency, causing the male voice to drop an octave and the female voice to drop 3 to 4 semitones. The register becomes low modal in men and high modal in women.^2^, ^3^, ^4^, ^5^

These voice changes are mostly due to hormonal factors, reflecting gender characteristics.

In boys the first signs of puberty are testicle enlargement and the growth of pubic hair, followed by axillary and facial hair. In girls initially breast enlarge and pubic hair develops; the first menstrual cycle begins with body growth.^6^

The main changes in the larynx are:

- increased anteroposterior distance;
- increased vocal fold length, width and thickness (growth in length is about 10.9 mm in men and 4.2 mm in women);
- lower positioning of the larynx relative to the spine.1,7

During this period voice fold lesions, specifically cysts and nodules, may undergo changes.^1^,^8^

In literature various authors have reported^8^, ^9^, ^10^ that cysts may rupture and become grooves or open cysts during the voice change period. Cysts are closed cavities located deep within vocal folds, usually in the surface layer of the lamina propria, and usually adhere to elastic and collagen vocal ligament fibers. During voice change, as the folds stretch, cysts tend to change into grooves or open cysts.

A groove is a longitudinal depression along the free edge of a vocal fold, which may cause atrophy of Reinke's space and adhesion between the mucosa and the vocal ligament. This condition affects vocal fold vibration. An open cyst is a fold of mucosa that opens as a pouch into the laryngeal lumen, and usually is fixed to deep layers.^10^

Cysts have well defined contours and a better surgical prognosis compared to the treatment of grooves and open cysts.1 Transformation of an encapsulated lesion after voice change, such as a cyst into a groove or open cyst, results in a lesion that is surgically more demanding.^1^,^8^ Along these lines it might be interesting to deal with vocal fold cysts in children if these changes do in fact take place. And what would be the impact on voice of these lesions after voice change? Do they always lead to worsening of voice?

The surgical prognosis of nodules, on the other hand, is more promising; they tend to be reabsorbed. Nodules may be characterized as a thickening of the free edge of both vocal folds along the limit between the anterior and middle portions; this is the region with the highest vibration amplitude and friction between vocal folds.1 According to Duprat,1 “after puberty nodules may regress completely.”

In fact many authors mention changes of vocal fold structural lesions after voice change,^11^,^12^ but there are no prospective published papers assessing children with these findings.

It is essential do define the lesions and alterations that may take place during voice change to establish a strategy for children with dysphonia.^4^,^11^,^13^ This paper aims to reassess children that were followed-up in our laryngology and voice outpatient department that presented cysts or nodules, and to detect voice complaints and alterations in vocal folds after voice change.

## OBJECTIVE

To assess the progression of structural changes of vocal folds such as nodules, cysts and grooves after voice change.

## MATERIAL AND METHOD

This study was approved by the Research Ethics Committee of the Santa Casa de Misericordia Sisterhood in Sao Paulo under the project number 273/04, on 1 October 2004.

We reviewed all of the videolaryngoscopic and nasofibroscopic exams of children presenting structural changes of vocal folds such as nodules, cysts or grooves, done between 1997 and 2002. Of these children, those that had already undergone voice change were invited to have the exam repeated.

Inclusion criteria for women were the presence of menses and the result of a questionnaire on voice change. Inclusion criteria in men were a similar questionnaire and an assessment by a speech therapist who investigated voice changes.

The exam of choice was videolaryngoscopy; nasofibroscopy was done only in those patients that had intense nausea and were thus unable to tolerate the exam.

The following equipment was used for videolaryngoscopy or nasofibroscopy:

- a Storz 10 mm 70^o^ rigid laryngeal telescope
- a Vision Sciences 3.5 mm flexible nasofibrolaryngoscope
- a Precision halogen 250W light source
- a Storz camera
- a 6-head Philips videocassette recorder
- a 20-inch LG 20 video monitor
- a Le Son microphone
- a Storz stroboscope

We assessed the original tape recorded before voice change and selected the best image; this image was sent to two professors/medical doctors who were specialized in laryngology and that belonged to the Otolaryngology Department. These professors jointly investigated the presence of bulges, thickening, vascular dysgenesis, grooves and fissures, and used stroboscopy to check symmetry and rigidity, and to describe the type of groove found. Lesions were classified as thickening or bulging due to difficulties in diagnostic definitions of these lesions in children. Thickening suggests nodules and bulging suggests cysts.

Patients were selected and recalled for a second assessment using the same protocol. Exams were recorded and evaluated by the same team. A questionnaire was applied to investigate current voice complaints. Dysphonia was characterized as intermittent or constant and related to voice abuse and/or other symptoms. Information was also gathered about the period of voice change, focusing on the effects of dysphonia in the periods before, during and after voice change. Discomfort was differentiated at being felt by the patient, parents, teachers or colleagues.

## RESULTS

Twelve patients were eligible for this study. One was excluded as the speech assessment showed a voice change vocal pattern.

Five patients were female (45.5%) and 6 patients were male (54.5%). Ages ranged from 13 to 17 years (mean = 14.54).

The questionnaire showed that 3 patients (27%) had complaints of dysphonia that was intermittent in 2 patients (66%) and constant in 1 patient (33%). Dysphonia was related to voice abuse in 2 patients (66%). Before voice change dysphonia caused discomfort in parents and patients in 81% of cases. Others reported complaints only from teachers and/or colleagues. During voice change only one mother of a patient complained of discomfort with her child's dysphonia.

After voice change, 3 patients continued with their complaint of dysphonia. In two cases dysphonia caused discomfort to the patients themselves; in one patient discomfort was reported by his girlfriend. (Graph 1)

Laryngoscopy before voice change revealed thickening that suggested vocal nodules in 5 cases (45.5%) and bulging that suggested cysts in 6 cases (54.5%). Bulging was on the left in 5 of 6 cases and bilateral in one patient. No vascular dysgenesis or groove type lesions were found. Laryngoscopy after voice change revealed thickening in 5 patients, being bilateral in 4 cases and only on the right in 1 case. Two patients presented grooves and no patient presented bulging or vascular dysgenesis (Graph 2)

The table below shows the number of patients with lesions before and after voice change, and their progression. Lesions shown in columns become the lesions shown

**Chart 1:**
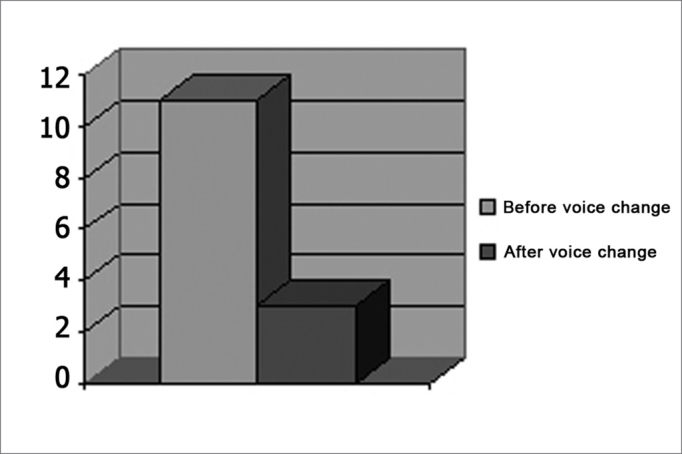
Frequency of complaints of dysphonia before and after voice change.

**Chart 2:**
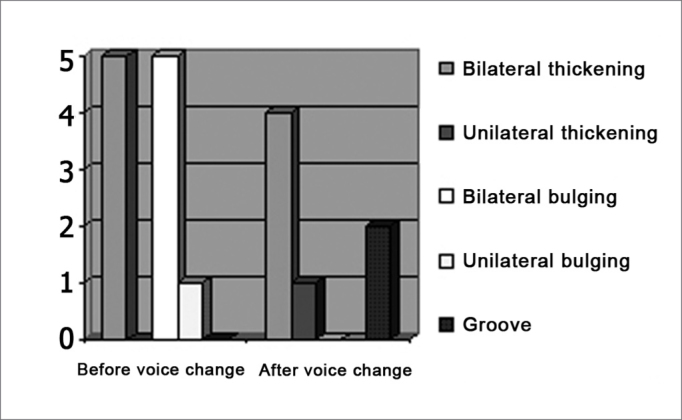
Frequency das vocal fold lesions before and after voice change.

on lines. (Table 1)

**Table 1:** Frequency of vocal fold lesions before and after voice change.

AFTER VOICE CHANGE					
		thickening	bulging	groove	no lesion
BEFORE VOICE CHANGE	thickening				5
	bulging	4		1	1

Stroboscopy revealed 6 cases of lesion-related rigidity in the period prior to voice change, of which 5 were associated with vibration asymmetry. After voice change there were 4 cases of rigidity associated with vibration asymmetry.

**Chart 3:**
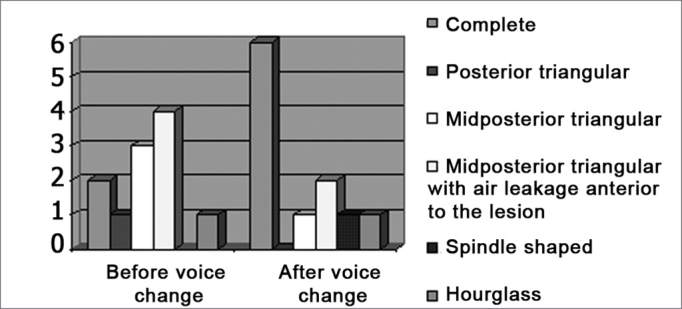
Frequency of the phonatory glottal fissure before and after voice change (mp: midposterior).

Nine patients presented fissures prior to voice change. These were one posterior fissure, one hourglass fissure, three mid-posterior triangular fissures, and four mid-posterior fissures with air leakage anterior to the lesion. After voice change one patient presented a mid-posterior triangular fissure, two patients had a mid-posterior fissure with air leakage anterior to the lesion, one had an hourglass fissure and one presented a spindle-shaped fissure. (Chart 3)

Table 2 shows how the changes occurred, where the fissures - shown on rows - become fissures shown in the columns. (Table 2)

**Table 2:** Frequency of phonatory glottal fissure before and after voice change.

AFTER VOICE CHANGE							
		A	B	C	D	E	F
Before Voice Change	A	1				1	
	B	1					
	C	1		1			1
	D	2			2		
	E						
	F	1					

A = complete closure

B = posterior triangular

C = midposterior triangular

D = midposterior triangular (with air leakage anterior to the lesion)

E = fusiform

F = hourglass

Of three patients that continued with complaints of dysphonia, one patient presented bilateral thickening with a bilateral fissure and a midposterior fissure with air leakage anterior to the lesion, the second patient presented bilateral thickening with a midposterior triangular fissure with air leakage anterior to the lesion, and the third patient presented a bilateral thickening with an hourglass fissure. Laryngoscopy before voice change demonstrated bulging on the left vocal fold with thickening of the right vocal fold, and stroboscopy showed asymmetry and rigidity in all three patients.

Eight patients had no complaints. Of these, 4 presented vocal fold lesions, as follows: bilateral thickening with a midposterior triangular fissure, a spindle-shaped fissure with rigidity to the left, thickening of the right vocal fold with rigidity, and a stroboscopically symmetrical bilateral groove.

## DISCUSSION

Our results are similar to those found in the literature we reviewed, which states that after voice change there is improvement in voice quality with a significant reduction in complaints of dysphonia. The assumption is that vocal fold stretching would alter their vibrating capability, modifying voice quality.

We found that only 3 of 11 patients persisted with the complaint of dysphonia after voice change.

Laryngoscopy also demonstrated that after voice change there was improved coaptation of vocal folds and a lower frequency of fissures. Once again, changes in laryngeal conformation are responsible not only for improved fold coaptation but also for changes in the slit pattern, noting that: the posterior slit disappeared; the incidence of mid-posterior slit with air leakage anterior to the lesion was reduced, although still the highest; the fusiform slit that initially did not exist appeared in one of the cases after voice change, and the posterior triangular and hourglass slits were found with similar frequency. Vocal fold changes due to lesions were significant slit-reducing and glottal proportion change factors, particularly in men; this may be related to vocal fold coaptation.

We found that laryngoscopy done before voice change showed vocal fold bulging suggesting cysts in all of the patients that complained of dysphonia after voice change. This finding is concordant with literature where various authors state that nodule-type lesions (thickening) disappear after voice change, while cystic-type lesions (bulging) tend to become grooves.

We found that two patients presented grooves after voice change, and both had laryngoscopic evidence of bulging suggesting cysts before voice change. Only one of these two patients reported dysphonia after voice change. These two cases suggest that the patient who persisted with dysphonia and a groove might have benefited from laryngeal microsurgery, as a cyst is easier to approach surgically than a groove formed after voice change. On the other hand, the patient presenting a groove but no complaint of dysphonia would not have benefited from surgery, as such a procedure could cause further adhesions. There are still many doubts about the behavior of these lesions.

Stroboscopy provided us with data on vocal fold rigidity; we found that patients persisting with dysphonia after voice change had vocal fold rigidity before voice change. Stroboscopy might be useful to detect regions of increased rigidity, supporting medical approaches of children with dysphonia.

Although many authors describe structural vocal fold changes after voice change, there are no published prospective studies assessing these children. Our paper adds to this debate, but further studies with larger samples, and studies that correlate laryngeal changes with anatomical and physiological development of the larynx during voice change are needed.

## CONCLUSIONS

After voice change, we observed:

- fewer complaints of dysphonia;
- lesions with thickening - suggest nodules - tend to disappear;
- lesions with bulging - suggest cysts - usually progress to grooves or lesions with thickening;
- stroboscopy: all patients that continued to complain of dysphonia after voice change had vocal fold rigidity before voice change;
- grooves tend to regress with time, with improvement in vocal fold coaptation.
